# Recent Advances in Histopathological and Molecular Diagnosis in Pheochromocytoma and Paraganglioma: Challenges for Predicting Metastasis in Individual Patients

**DOI:** 10.3389/fendo.2020.587769

**Published:** 2020-10-27

**Authors:** Yuto Yamazaki, Xin Gao, Alessio Pecori, Yasuhiro Nakamura, Yuta Tezuka, Kei Omata, Yoshikiyo Ono, Ryo Morimoto, Fumitoshi Satoh, Hironobu Sasano

**Affiliations:** ^1^ Department of Pathology, Tohoku University Graduate School of Medicine, Sendai, Japan; ^2^ Division of Clinical Hypertension, Endocrinology and Metabolism, Tohoku University Graduate School of Medicine, Sendai, Japan; ^3^ Division of Pathology, Faculty of Medicine, Tohoku Medical and Pharmaceutical University, Sendai, Japan; ^4^ Division of Nephrology, Endocrinology, and Vascular Medicine, Tohoku University Hospital, Sendai, Japan

**Keywords:** adrenal, pheochromocytoma, paraganglioma, genotype, pathology, SDHB, PASS, GAPP

## Abstract

Pheochromocytomas and paragangliomas (PHEO/PGL) are rare but occasionally life-threatening neoplasms, and are potentially malignant according to WHO classification in 2017. However, it is also well known that histopathological risk stratification to predict clinical outcome has not yet been established. The first histopathological diagnostic algorithm for PHEO, “PASS”, was proposed in 2002 by Thompson et al. Another algorithm, GAPP, was then proposed by Kimura et al. in 2014. However, neither algorithm has necessarily been regarded a ‘gold standard’ for predicting post-operative clinical behavior of tumors. This is because the histopathological features of PHEO/PGL are rather diverse and independent of their hormonal activities, as well as the clinical course of patients. On the other hand, recent developments in wide-scale genetic analysis using next-generation sequencing have revealed the molecular characteristics of pheochromocytomas and paragangliomas. More than 30%–40% of PHEO/PGL are reported to be associated with hereditary genetic abnormalities involving > 20 genes, including *SDHXs, RET, VHL, NF1, TMEM127, MAX*, and others. Such genetic alterations are mainly involved in the pathogenesis of pseudohypoxia, *Wnt*, and kinase signaling, and other intracellular signaling cascades. In addition, recurrent somatic mutations are frequently detected and overlapped with the presence of genetic alterations associated with hereditary diseases. In addition, therapeutic strategies specifically targeting such genetic abnormalities have been proposed, but they are not clinically applicable at this time. Therefore, we herein review recent advances in relevant studies, including histopathological and molecular analyses, to summarize the current status of potential prognostic factors in patients with PHEO/PGL.

## Introduction

Pheochromocytomas (PHEOs)/paragangliomas (PGLs) or PPGLs are not only oncological diseases due to their invasive or metastatic properties, but also life-threatening endocrinological disorders associated with medical therapy resistant hypertension due to catecholamine excess (1–4). Differentiation between “PHEOs” and “PGLs” is defined based on the sites of the primary lesion as follows; PHEOs are derived from chromaffin cells in the adrenal medulla, and PGLs from sympathetic or parasympathetic paraganglion cells located in extra-adrenal tissues (5).

Distant metastasis is detected in 5%–20% of PHEOs, and relatively higher in PGLs, ranging from 15% to 35% (6–9). The five-year survival rate of metastatic disease has been reported to be approximately 50% or less (10–12). However, it is difficult to predict metastatic potential based on histopathological findings alone, and none of the previously proposed histopathological scoring systems can reach the levels of accurate metastasis prediction. Therefore, all PPGLs were proposed to have malignant potential according to the WHO classification in 2017, because of the absence of hallmark diagnostic markers (5).

In contrast, recent developments in molecular analysis have clarified the genetic landscape or characteristics of PPGLs, which could reflect the risks of metastatic potential (1–4, 6). The results of those studies revealed a higher incidence of genetic abnormalities associated with hereditary diseases, spanning more than 20 relevant genes in > 40% of all cases (1–4, 6). Among the genes above, the presence of *SDHX* mutations is reported to increase the risks of developing aggressive disease behavior by altering intracellular metabolism, especially the tricarboxylic acid (TCA) cycle (4, 13–17).

In this review, we therefore summarized the previously proposed histopathological/clinicopathological scoring systems, including their limitations for predicting the metastatic potential of the disease, and pitfalls when interpreting the findings. In addition, the clinical significance of recently reported genetic abnormalities and genotype-phenotype associations are also summarized.

## Genetic Abnormalities in PPGLs

PPGLs were previously called “10%-diseases” associated with hereditary disorders. However, recent developments in genetic analysis using next-generation sequencing and large-scale integrated analysis by The Cancer Genome Atlas (TCGA) database has identified a much larger number of relevant genetic abnormalities (6, 18). The prevalence of PPGLs associated with hereditary diseases involves approximately 40% of all patients (6). Pathogenic variants with genetic alterations in relevant genes are generally exclusive to each other, but it is also true that somatically mutated driver genes are involved in further development of PPGLs in a minor population with germline mutations (6), which is considered unique to this tumor. In addition, comprehensive genetic analysis by Fishbein et al. further demonstrated that 27% of PPGLs have germline mutations, 39% somatic mutations (with 5%–10% overlap with germline mutations), 7% gene fusions, and 89% copy number alterations (6). PPGLs are sub-classified into three different groups, according to their genotype-related pathophysiology (4, 6, 19–21). The most prevalent subtype is the “pseudohypoxia type”, with genetic alterations in *SDHX* families, *FH*, *VHL*, and *EPAS1* (13–17, 22–27). The second is the “Wnt-signal type” associated with somatic alterations in genes involved in Wnt-signaling pathways, including *CSDE1* mutation and *MAML3* gene transfusion (6, 28). The third is the “kinase signal type” with genetic alterations involving *RET*, *NF1*, *MAX*, and *TMEM127*, and which is frequently associated with MEN2 (multiple endocrine neoplasia type 2) gene abnormalities (4, 6, 29–35). In addition, a fourth group was also recently proposed as a cortical admixture subtype, although the detailed features involved have remained uncertain compared to the three major subtypes indicated above (6). Therefore, in this paper, individual genotypes and their pathophysiological characteristics are briefly reviewed. Previously reported genetic alterations associated with PPGLs are also summarized in **Table 1**.

**Table 1:** Previously identified mutated driver genes associated with PPGLs.

Type	Gene	Conding Protein	Chromosome location	Germiline or Somatic	Predominant tumor site	Contribution to metastatic potential	Associated hereditary diseases	Reference
1	*SDHA*	Succinate Dehydrogenase Complex Flavoprotein Subunit A	5p15.33	Germline	PGL>PHEO	Low	Famlilial PGL type 5	(6, 13),
1	*SDHB*	Succinate Dehydrogenase Complex Iron Sulfur Subunit B	1p36.13	Germline	PGL>PHEO	Intermediate	Famlilial PGL type 4	(14)
1	*SDHC*	Succinate Dehydrogenase Complex Subunit C	1q23.3	Germline	PGL>>PHEO	Very low	Famlilial PGL type 3	(15)
1	*SDHD*	Succinate Dehydrogenase Complex Subunit D	11q23.1	Germline	PGL>PHEO	Low	Famlilial PGL type 1	(6, 16)
1	*SDHAF2*	Succinate Dehydrogenase Complex Assembly Factor 2	11q12.2	Germline	PGL>>PHEO	Very Low	Famlilial PGL type 2	(17, 36)
1	*FH*	Fumarate Hydratase	1q43	Germline	PHEO, PGL	Low	FH-deficient HLRCC (Hereditary leiomyomatosis and renal cell carcinoma)	(37)
1	*VHL*	Von Hippel-Lindau Tumor Suppressor	3p25.3	Germline	PHEO>PGL	Low-Intermediate	Von-Hippel-Lindau disease	(6, 25),
1	*EPAS1 (HIF2A)*	Endothelial PAS Domain Protein 1	2p21	Germline, Somatic	PHEO, PGL	Low-Intermediate	Pacak-Zhuang syndrome	(6, 26, 27)
1	*EGLN1 (PHD1)*	Egl-9 Family Hypoxia Inducible Factor 1	1q42.2	Germline	PHEO, PGL	Not characterized	Polycythemia	(6, 38)
1	*EGLN2 (PHD2)*	Egl-9 Family Hypoxia Inducible Factor 2	19q13.2	Germline	PHEO, PGL	Not characterized	Polycythemia	(38)
1	*MDH2*	Malate Dehydrogenase 2	7q11.23	Germline	PHEO, PGL	Not characterized	Not characterized	(23, 39),
1	*SLC25A11*	Solute Carrier Family 25 Member 11	17p13.2	Germline	PGL	Low-Intermediate	Not characterized	(40)
1	*DLST*	Dihydrolipoamide S-Succinyltransferase	14q24.3	Germline	PHEO, PGL	Not characterized	Not characterized	(41)
1	*DNMT3A*	DNA Methyltransferase 3 Alpha	2p23.3	Germline, Somatic	PHEO, PGL	Not characterized	Acute Myeloid Leukemia (AML) (42)	(43)
1	*GOT2*	Glutamic-Oxaloacetic Transaminase 2	16q21	Germline	PHEO, PGL	Not characterized	Not characterized	(44)
2	*CSDE1*	Cold Shock Domain Containing E1	1p13.2	Somatic	PHEO, PGL	Not characterized	―	(6)
2	*MAML3*	Mastermind Like Transcriptional Coactivator 3	4q31.1	Somatic, Transfusion	PHEO, PGL	Low-Intermediate	―	(6, 28),
3	*KIF1B*	Kinesin Family Member 1B	1p36.22	Germline	PHEO?	Not characterized	Ganglioneuroma, leiomyosarcoma, lung adenocarcinoma, neuroblastoma, ganglioneuroma	(45)
3	*RET*	Proto-Oncogene Tyrosine-Protein Kinase Receptor Ret	10q11.21	Germline, Somatic	PHEO>>PGL	Low	Multiple endocrine neoplasia type 2	(6, 29–31),
3	*NF1*	Neurofibromin 1	17q11.2	Germline, Somatic	PHEO>PGL	Low	Nuerofibromatosis type 1	(6, 29–32),
3	*MAX*	MYC Associated Factor X	14q23.3	Germline	PHEO>PGL	Low	Familial PCC	(6, 34, 35),
3	*TMEM127*	Transmembrane Protein 127	2q11.2	Germline	PHEO>PGL	Low	Familial PCC	(6, 33),
3	*HRAS*	GTPase HRas	11p15.5	Somatic	PHEO?	Not characterized	―	(6)
3	*BRAF*	Serine/Threonine-Protein Kinase B-Raf	7q34	Somatic	PHEO, PGL	Not characterized	―	(6)
Others	*MEN1*	Menin 1	11q13.1	Germline	PHEO, PGL	Not characterized	Multiple endocrine neoplasia type 1	(46)
Somatic	*IRP1*	Iron Regulatory Protein 1	9p21.1	Somatic	PHEO, PGL	Not characterized	―	(47)
Somatic	*SETD2*	Histone-Lysine N-Methyltransferase SETD2	3p21.31	Somatic	PHEO, PGL	Low-Intermediate	―	(6, 18, 48),
Somatic	*FGFR1*	Fibroblast Growth Factor Receptor 1	8p11.23	Somatic	PHEO, PGL	Not characterized	―	(6, 49),
Somatic	*MET*	Hepatocyte Growth Factor Receptor	7q31.2	Somatic	PHEO, PGL	Not characterized	―	(50)
Somatic	*TP53*	Cellular Tumor Antigen P53	17p13.1	Somatic, Germline	PHEO, PGL	Not characterized	Li-Fraumeni Syndrome	(6)
Somatic	*ARNT*	Aryl Hydrocarbon Receptor Nuclear Translocator	1q21.3	Somatic	PGL	Not characterized	―	(6)
Somatic	*MYO5B*	Myosin VB	18q21.1	Somatic	PHEO, PGL	Not characterized	―	(51, 52),
Somatic	*MYCN*	N-Myc Proto-Oncogene Protein	2p24.3	Somatic	PHEO, PGL	Not characterized	―	(51)
Somatic	*VCL*	Vinculin	10q22.2	Somatic	PHEO, PGL	Not characterized	―	(51)
Somatic	*KMT2D*	Histone-Lysine N-Methyltransferase 2D	12q13.12	Somatic	PHEO, PGL	Not characterized	―	(53)
Somatic	*TERT*	Telomerase Reverse Transcriptase	5p15.33	Somatic	PHEO, PGL	Low-Intermediate	―	(54–57),
Somatic	*ATRX*	Transcriptional regulator ATRX	Xq21.1	Somatic	PHEO, PGL	Low-Intermediate	―	(6, 36, 58–60)
Somatic	*IDH1*	Isocitrate Dehydrogenase (NADP(+)) 1	2q34	Somatic	PHEO, PGL	Not characterized	―	(6, 59)
Somatic	*IDH2*	Isocitrate Dehydrogenase (NADP(+)) 2	15q26.1	Somatic	PHEO, PGL	Not characterized	―	(61)
Somatic	*H3F3A*	H3 Histone Family Member 3A	1q42.12	Somatic	PHEO, PGL	Not characterized	―	(50)

Type 1 Pseudohypoxia type, Type 2: Wnt signal type, Type 3: Kinase signal type.

In addition to more than 20 genes with germline mutations, recently detected genes with somatic variants are also summarized in this table. Some genes with somatic variants were classified into three previously known types if the detailed function of the mutated genes was clarified.

### “Pseudohypoxia Type”

“Pseudohypoxia type” is the most prevalent phenotype in PPGLs, and the great majority of genetic abnormalities involving this phenotype have been detected in genes involved in the TCA cycle, including *SDHX* family, *FH*, *VHL*, *EPAS1, SLC25A11*, and others (13–17, 22–27). Chromaffin cells are physiologically involved in oxidative metabolism status, with abundant aerobic respiration by mitochondria synthesizing ATP by activating the TCA cycle. However, genetic alterations in genes encoding catalyzing enzymes involved in the TCA cycle, such as succinate dehydrogenase, are known to result in loss of their physiological functions. These altered genes subsequently promote anaerobic metabolism by tumor cells, shifting ATP resources from the TCA cycle into the system of metabolic glycolysis (62–64). These alterations in intracellular metabolism eventually result in degradation of chromatin remodeling, reactive oxygen species production, and DNA methylation (62–66). These intracellular changes also enable tumor cells to efficiently synthesize ATP, although the amounts of ATP synthesized from glycolysis per reaction does not reach the same levels as those from the TCA cycle (62–66). This phenomenon has attracted considerable interest because of its possible associations with Warburg effects detected in some neoplastic cells (65, 66). Therefore, sub-typing based on intracellular metabolism in PPGLs has also been proposed. Some clinical studies exploring the ability of glucose absorption in PPGLs by FDG-PET imaging have been reported, and are proposed to be practically useful as a noninvasive diagnostic tool, especially for detecting pseudohypoxic phenotypes of tumors, and those manifesting potentially malignant behavior over their clinical course (67, 68).

### “Wnt-Signal Type”

The “Wnt-signal type” is known as the most prevalent phenotype among sporadic PPGLs, with somatic alterations to driver genes (4, 6). Wnt-/Shh-related pathways are widely reported to be involved in cell proliferation in various types of diseases (69, 70). The activation of Wnt-related signals is not necessarily specific for PPGLs, but the presence of this particular type of genetic abnormality has been reported to result in relatively frequent distant metastasis or recurrence, especially in cases involving *MAML3* gene fusions (6). Somatic mutations of the *CSDE1* gene and transfusion of *MAML3* are both classified as exhibiting this phenotype. *CSDE1* frameshift and splice-site mutations have been reported in a minor population of PPGLs with previously known germline mutations, including *VHL*, *NF1*, and *RET* (6). These *CSDE1* genetic alterations result in loss-of-function (6). *CSDE1* is well known in regulating translation initiation, apoptosis, RNA stability, and differentiation/development of neuronal tissue (71, 72). The functional roles of mutated variants of *CSDE1* were also previously validated by microarray analysis using mouse embryonic stem cells (73, 74).

PPGLs with *MAML3* gene fusions are reported to be associated with a higher prevalence of metastatic diseases, frequently in conjunction with SDH loss (6, 28). Comprehensive genetic analysis revealed that the *UBTF-MAML3* fusion gene activates *Wnt-Shh* signaling, but only a small number of studies have investigated the clinical significance of this chimeric fusion gene (6, 28). Therefore, the detailed underlying mechanisms, as well as their prevalence, have not been thoroughly studied, and further investigations are warranted.

### “Kinase Signal Type”

The “kinase signal type” is associated with systemic hereditary diseases such as MEN2A/2B (*RET* mutation) and neurofibromatosis type 1 (*NF1* mutation) (29–32). Familial PHEOs with *TMEM127* or *MAX* mutations are also categorized into this subtype (33–35). Among them, the gain-of-function caused by *RET* gene mutation has been studied in the most detail. *RET* encodes a transmembrane receptor tyrosine kinase involved in the development of the neural crest. *RET* mutations detected in MEN2A are reported to cause homodimerization, which subsequently activates PI3K-AKT, RAS, p38-MAPK, and JUN N-terminal kinase pathways in a ligand-independent manner, promoting abnormal cell proliferation (75–77). Recently, somatic mutations detected involving *FGFR1*, *NF1*, *BRAF*, *HRAS*, and others have also been reported to contribute to the activation of the relevant pathways indicated above (6). However, the underlying mechanisms involving the kinase signaling pathway remain unknown, especially whether these pathways possibly interact with the downstream pathways of other subtypes.

### Others (Somatic Abnormalities)

With the exception of three major subgroups, multiple somatic genetic abnormalities have been reported, involving *IRP1* (47), *SETD2* (6, 18, 48), *FGFR1* (6, 49), *MET* (50), *TP53* (6), *ARNT* (6), *MYO5B* (51, 52), *MYCN* (51), *VCL* (51), *KMT2D* (53), *TERT* (54–57), *ATRX* (6, 57–59), *IDH1* (6, 58), *IDH2* (36), and *H3F3A* (50). However, it is also true that majority of newly reported somatic gene abnormalities are detected in only a minor proportion of patients with PPGLs. Among these somatic gene abnormalities, aberrant telomere maintenance mechanism (TMM), which is caused by TERT (telomerase reverse transcriptase) structural rearrangement, genetic abnormalities, and *ATRX* mutations, has been reported to be associated with poor clinical outcomes in patients (54–57). Structural rearrangement of TERT has also been reported to result in its over-expression as a result of the placement of enhancers proximal to the *TERT* promoter (56). The presence of somatic mutations detected in the *TERT* promoter is not necessarily concordant with TERT overexpression, but a specific hot-spot, C228T, is reported to be associated with adverse clinical outcomes in patients (57, 78). However, its cross-interaction with *SDHX*-related pseudohypoxic pathways cannot be ruled out.

## Challenges of Predictive Clinicopathological/Histopathological Scoring Systems for Malignant Behavior/Metastasis in PPGLs

Histopathological risk stratification, or discerning malignancy, in PPGL patients is very challenging and is generally considered one of the most difficult differential diagnoses in the field of surgical pathology. Several histopathological scoring systems have been proposed, including PASS and GAPP scores, but it is also true that those above could by no means precisely predict the clinical outcome and/or the degree of aggressive clinical behavior in PPGL patients (5, 79–81). As a basis for these two established representative histological scoring systems, several combined scoring systems with genetic abnormalities and immunohistochemical findings have also been recently proposed, such as M-GAPP (Modified-GAPP) score (82), ASES (Age, Size, Extra-adrenal location, and Secretory type) score (83) and COPPs (Composite Pheochromocytoma/paraganglioma Prognostic score) (84). However, further investigations are needed to clarify the practical value of such systems in discerning the clinical behavior of patient tumors.

Therefore, in this section, previously proposed histopathological/clinicopathological scoring systems and the recent validation studies of these systems were covered to clarify the usefulness and limitations of histopathological findings to predict the clinical behavior of tumors, as well as the potential pitfalls involving interpretation of such findings with high inter-/intra-observer variation by both pathologists and clinicians.

### PASS (Pheochromocytoma of the Adrenal Gland Scale Score)

**Figure 1:**
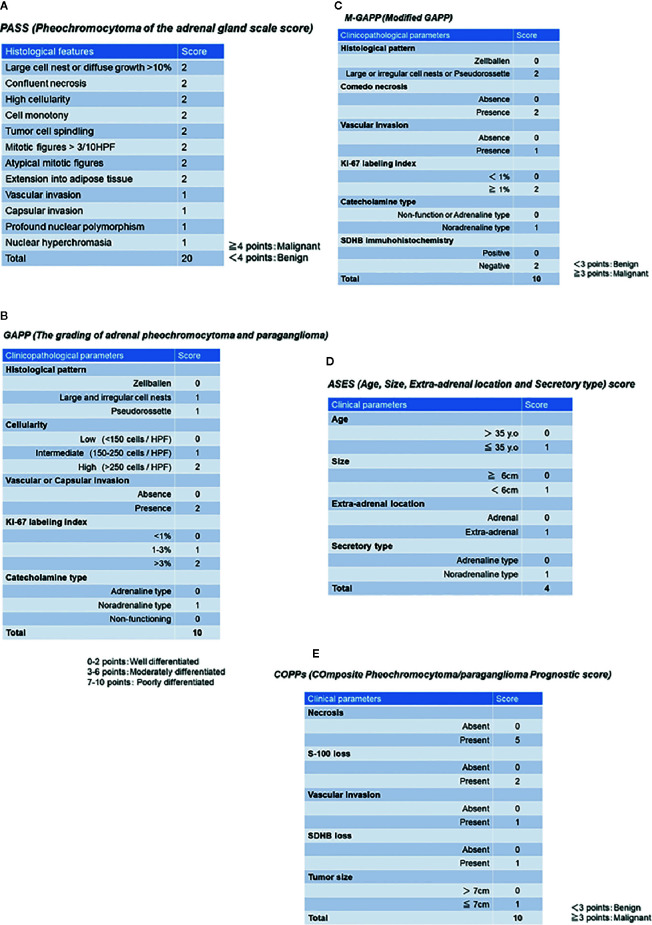
Previously proposed histopathological/clinicopathological scoring system. **(A)** PASS (Pheochromocytoma of the adrenal gland scale score). **(B)** GAPP (Grading of adrenal pheochromocytoma and paraganglioma). **(C)** M-GAPP (Modified GAPP). **(D)** ASES (Age, Size, Extra-adrenal location and Secretory type) score. **(E)** COPPs (Composite Pheochromocytoma/paraganglioma Prognostic score).

PASS was the first histopathological scoring system proposed by the group of Armed Forces Institute of Pathology led by Thompson in 2002, and this system was composed of twelve findings based on histological features as follows (summarized in **Figure 1A**): 1) large cell nests or diffuse growth of >10%, 2) central or confluent tumor necrosis, 3) high cellularity, 4) cell monotony, 5) tumor cell spindling (even if focal), 6) mitotic figures >3 figures/10 high power fields, 7) atypical mitotic figure(s), 8) extension into adipose tissue, 9) vascular invasion, 10) capsular invasion, 11) profound nuclear polymorphism, 12) and nuclear hyperchromasia (79). Tumors with 4 points or more were proposed to be associated with a high prevalence of distant metastasis, and those with less than 4 points considered as benign (never metastatic) (79). Of particular note, the use of PASS in extra-adrenal PGLs was limited because this particular scoring system was designed only for PHEOs, and included those criteria only applicable to intra-adrenal tumors such as extension into adipose tissue (81).

After the proposal of PASS, several validation studies were reported in the literature (82, 85–87). The presence of relatively high inter-/intra-observer variation has been reported in the confirmatory studies indicated above. Among those 12 histological features above, the presence of capsular and vascular invasion, extension into adipose tissue, and atypical mitosis could reach relatively high inter-observer concordance in > 80% of the examined cases (88). However, the histological features of high cellularity, profound nuclear polymorphism, and nuclear hyperchromasia resulted in low inter- and intra- observer concordance in their interpretation, even among pathologists with sufficient experience and knowledge in this field (88). Furthermore, it is also pivotal to note that the gradients of scoring points of individual histological features did not necessarily match the degree of inter-/intra-observer variation (88). Scoring systems based only on morphological or histological findings could become more subjective and, therefore, some studies employing combined PASS and genetic abnormality, as well as immunohistochemistry, have been proposed in recent years in order to overcome potential disadvantages or pitfalls of the system, as described above.

### GAPP Score (Grading of Adrenal Pheochromocytoma and Paraganglioma) and M-GAPP (Modified GAPP)

The GAPP score was proposed by Kimura et al. in 2014 and required not only morphological findings, but also clinically proven catecholamine-producing types and proliferative ability of tumor cells by Ki-67 (MIB-1) labeling index (LI), in contrast to PASS, which could be performed only on hematoxylin-eosin stained tissue slides. This GAPP scoring system classified PPGLs into three different grades: well- (0-2 points), moderately (3-6 points), and poorly differentiated (7-10 points) PPGLs (80). The details of this scoring system are summarized in **Figure 1B**. The five-year survival rates of these three groups are 100% (well-differentiated), 66.8% (moderately differentiated), and 22.4% (poorly differentiated) (80). GAPP has been used in some diagnostic pathology laboratories, but several limitations or pitfalls have been raised regarding its clinical utility (4, 5, 81). In particular, MEN2A-associated PPGLs are over-diagnosed by both PASS and GAPP in predicting the potential malignant behavior of tumors (85). MEN2A-associated PPGLs rarely metastasize, although large cell nests or diffuse growth patterns (MEN2A-associated: 77% vs. benign: 30%, malignant: 90%) and increased Ki-67 LI of > 3% (MEN2A-associated: 31% vs. sporadic: 11%) are frequently detected in such cases, which result in high scores (85). In addition, the original GAPP system did not include finding regarding *SDHX* status (80). Therefore, Koh et al. subsequently proposed a modified GAPP score, modifying the gradient of the scoring points, and added the findings of SDHB immunohistochemistry (82). The details of M-GAPP are summarized in **Figure 1C**. The sensitivity of GAPP and M-GAPP is relatively high, while their specificity only reaches 50%–60% in terms of predicting distant metastasis in PPGL patients (82). The area under the curve (AUC) of these scoring systems resulted in 0.822 for M-GAPP, 0.728 for GAPP, and 0.753 for PASS (82), and there were no differences among the predictive values for patients. Therefore, other clinicopathological factors such as tumor size or patient age should be considered when determining the malignant potential of PPGLs. Further improvements in histopathological evaluation are warranted to more precisely predict the malignant potential of tumors.

### ASES (Age, Size, Extra-Adrenal Location, and Secretory Type) Score

ASES (Age, Size, Extra-adrenal location and Secretory type) scoring was recently proposed by Cho et al. in 2018 (83). They performed a retrospective analysis using a relatively large number of cases, including 333 PPGLs (83). In contrast to other histopathological predictive models, ASES is entirely composed of only 4 clinical parameters (**Figure 1D**). The AUC to predict malignant behavior is reported to be 0.735 (88), and the practical advantages of using this scoring system includes no requirement for surgical specimens, which could apply this scoring system to all PPGLs, regardless of clinical stage (83). However, the sensitivity and specificity of these histology-based scoring systems remain unknown.

### COPPs (Composite Pheochromocytoma/Paraganglioma Prognostic Score)

COPPs (Composite Pheochromocytoma/paraganglioma Prognostic score) was recently proposed by Pierre et al. in 2019, integrating morphological features and immunohistochemical findings of S-100 and SDHB (84). They examined a total of 147 PPGLs and performed multivariate analysis, including incorporation of the morphological features listed in PASS, immunohistochemical findings of S-100, Ki-67, and MCM6, clinicopathological factors (tumor size, age, and hypertension) and genotype (84). Finally, COPPs were defined according to the following criteria: three clinicopathological parameters (tumor size > 7 cm, necrosis, and vascular invasion), loss of S-100 immunoreactivity (loss of intervening sustentacular cells), and loss of SDHB immunoreactivity (suggesting *SDHB* mutation) (84) (**Figure 1E**). When compared with previously proposed scoring systems, COPPs could provide a high AUC to predict potential metastasis in patients (sensitivity: 100%, specificity: 94.7%) (84). However, prospective validation studies involving COPPs have not been reported, and not all of the parameters proposed in this scoring system are readily available in clinical practice. Thus, COPPs could not reach the levels suitable for practical usage in current clinical settings and awaits validation.

## Practical Immunohistochemical (IHC) PPGL Markers

In addition to the above previously proposed clinicopathological scoring systems, several immunohistochemical (IHC) markers have also been reported in the literature to be able to differentiate metastatic from non-metastatic PPGLs. In this paper, the practical usefulness of IHC and its limitations and pitfalls in daily clinical settings are summarized.

### Conventional Markers

SDHB IHC has been employed to detect *SDHB* gene mutations with relatively high concordance (sensitivity: 100% [95% CI: 87%–100%], specificity: 84% [95% CI: 60%–97%]) as demonstrated by the total absence of immunoreactivity, with positive immunoreactivity in endothelial cells as a positive IHC control (89). However, it is pivotal to note that interpretation of SDHB IHC is sometimes difficult because of the presence of false-negative findings, caused by various pre-analytical factors such as inappropriate fixation, which results in various staining patterns, including potential false-negative findings (89, 90). In particular, patterns of SDHB immunoreactivity with a complete absence, or weak but diffuse dot-like cytoplasmic staining patterns were detected in *SDHB*-mutated PPGLs (90). Therefore, confirmatory genetic analysis is practically mandatory for cases with equivocal immunoreactivity.

Both S-100 and Ki-67 are well-known and widely used markers for evaluation of the malignant potential of PPGLs (80, 81, 84). S-100 is generally immunolocalized in sustentacular cells surrounding tumor cells (91). Absence or attenuation of S-100 immunoreactivity (sustentacular cells) is generally considered to reflect diffuse growth patterns that deviate from the structure of Zellballen, possibly resulting in the aggressive clinical behavior of tumors (84, 91). S-100 positive sustentacular cells have recently been reported as non-neoplastic cells because SOX-10 and SDHB are both positive only in sustentacular cells in the cases of *SDHB*-mutated PPGLs (91). However, detailed characterization of sustentacular cells remains to be conducted.

The Ki-67 LI is also listed as one of the parameters in GAPP and M-GAPP. However, it is also important to note that Ki-67 LI is generally low (< 3%) in > 80% of PPGLs, and its intratumoral heterogeneity is also marked (80–82). In addition, the guidelines to obtaining Ki-67 LI, such as whether counting should be performed in “hot spots” or “averages”, have not necessarily been standardized, and inter-observer or -laboratory differences in Ki-67 LI results might be unavoidable.

Thus, these IHC markers are marginally useful for predicting the clinical behavior of tumors, but none of the previously proposed IHC markers are by no means independent predictive markers in patients.

### Catecholamine-Synthesizing Enzymes

In addition to broadly used IHC markers, analyses of hormonal activities and IHC analysis of catecholamine-synthesizing enzymes such as tyrosine hydroxylase (TH), dopamine beta hydroxylase (DBH), dopa decarboxylase (DDC) and phenylethanolamine N-methyltransferase (PNMT) have also been reported in the literature. The expression profiles of these enzymes do not only characterize the secretory phenotypes of norepinephrine or epinephrine, but also reflect differentiation of the tumor cells in PPGLs (80, 92). PNMT catalyzes the final step of catecholamine biosynthesis from norepinephrine into epinephrine. Of particular interest, pseudohypoxic PPGLs are generally negative for PNMT, and have silent clinical and hormonal phenotypes, which could delay therapeutic intervention in such patients (93). Fukaya et al. reported that lower DDC immunoreactivity was detected in poorly differentiated PPGLs, histologically representing confluent necrosis, diffuse growth, nuclear polymorphism, and tumor cell spindling (94). Therefore, it is considered worthwhile to incorporate IHC analysis of these four catecholamine-producing enzymes into routine clinical practice in institutions treating relatively large volumes of patients with PPGLs because antibodies against all four enzymes used for IHC are commercially available (94).

### Newly Proposed Markers

In addition to the classical markers above, several relatively unique IHC markers have recently been proposed for predicting the presence of distant metastasis in PPGLs. Deng et al. reported lower immunoreactivity of Snail, Galectin-3, and IGF-1R in benign PHEOs without local invasion and distant metastasis, based on a study of 226 PPGL cases (95). Leijon et al. immunolocalized SSTR (somatostatin receptor) family as a potential prognostic factor or a therapeutic target, and reported that 71.4% (10/14) of cases of metastasized PPGLs abundantly expressed SSTR2 (96). Among them, different immunoprofiles were detected between metastasized PGLs and PHEOs (PGLs: 100% (9/9 cases), PHEOs: 20% (1/5 cases). In contrast, SSTR4 and SSTR5 were IHC-negative in the majority of the cases examined, and both SSTR1 and SSTR3 were divergent and independent of *SDHX* deficiency, as well as the presence of metastases (96). However, the usefulness of somatostatin analogs in the treatment of patients with PPGLs has not been established, and the clinicopathological value of SSTR IHC should be validated by further studies. Surrogate markers associated with tumor immune microenvironmental factors have been studied recently, especially PD-1/PD-L1 in PPGLs (97, 98). Guo et al. examined PD-L1 immunoreactivity in 77 PPGL cases using an anti-PD-L1 antibody (clone E1L3N) and reported that 59.74% (46/77 cases) of PPGLs were IHC-positive for PD-L1, with high co-efficiency of Ki-67 LI, as well as the presence of hypertension (97). On the other hand, Pinato et al. examined 100 PPGL cases using the same anti-PD-L1 antibody (clone E1L3N) and anti-PD-L2 antibody (polyclonal) (98). They reported that PD-L1 was IHC-positive in 18% (18/100 cases) and PD-L2 in 16% (16/100 cases) of PPGLs, respectively (98). Of particular interest, PD-L2 immunoreactivity in tumor cells was significantly correlated with overall survival of patients in their study (98). The presence of PD-L1 immunoreactivity in tumor cells could potentially indicate the utility of immune-checkpoint inhibitors, but standardization of histopathological evaluation of such markers, as well as unification of IHC antibody clones, are mandatory before various immune checkpoint inhibitors can be used therapeutically in PPGLs. In addition, few studies have reported histopathological surrogate markers of the tumor-immune microenvironment in PPGLs, and the clinical therapeutic efficacy of immune-checkpoint inhibitors remains unknown.

In summary, with a possible exception of SDHB, IHC-based analysis was less predictive than genetic analysis and past clinical history of the relevant hereditary diseases, and none of the above could be an independent predictive marker or a therapeutic target molecule. Therefore, future clinical trials as well as investigations of novel therapeutic targets are warranted in PPGLs.

## Summary

Recent advances in genetic and molecular characterization have classified PPGLs into subgroups based on their genotype-related pathophysiology. These genetic abnormalities are frequently detected in approximately 40% of PPGLs, far more than proposed over the past decades. Among them, *SDHX* mutations are the most frequently detected, resulting in pseudohypoxic status of tumor cells and which correlate with patient clinical outcomes, especially in detecting metastatic potential. Several histopathological and clinicopathological scoring systems have been proposed, but it is still challenging for diagnostic pathologists to predict malignant behavior based on histopathological findings of resected specimens alone, in contrast to other tumors such as adrenocortical neoplasms. Therefore, comprehensive scoring systems, combined with histopathological findings, genotyping, IHC, hormonal activities (metabolic phenotypes), the sites of involvement, and other clinical parameters have recently been proposed in the literature. However, none of the scoring systems reported could reach the necessary levels of practical usage or incorporation into clinical guidelines with high accuracy. In addition, no surrogate markers of specific therapy in patients with PPGL have been identified. Further investigations are required to clarify detailed pathophysiology of PPGLs, as well as more precise patient risk stratification.

## Author Contributions

All authors contributed to the article and approved the submitted version.

## Funding

This work was supported by a Health Labour Sciences Research Grant (No. H29-Nanji-Ippan-046).

## Conflict of Interest

The authors declare that the research was conducted in the absence of any commercial or financial relationships that could be construed as a potential conflict of interest.

## References

[1] Neumann HPH Young WF Jr Eng C Pheochromocytoma and Paraganglioma. N Engl J Med (2019) 381:552–65. 10.1056/NEJMra1806651 31390501

[2] Alrezk R Suarez A Tena I Pacak K Update of Pheochromocytoma Syndromes: Genetics, Biochemical Evaluation, and Imaging. Front Endocrinol (Lausanne) (2018) 9:515. 10.3389/fendo.2018.00515 30538672 PMC6277481

[3] Nölting S Ullrich M Pietzsch J Ziegler CG Eisenhofer G Grossman A Current Management of Pheochromocytoma/Paraganglioma: A Guide for the Practicing Clinician in the Era of Precision Medicine. Cancers (2019) 11:1505. 10.3390/cancers11101505 PMC6827093 31597347

[4] Crona J Taïeb D Pacak K New Perspectives on Pheochromocytoma and Paraganglioma: Toward a Molecular Classification. Endocr Rev (2017) 38:489–515. 10.1210/er.2017-00062 28938417 PMC5716829

[5] WHO classification Endocrine Tumor. IARC (International Agency for Research on Cancer) (2017).

[6] Fishbein L Leshchiner I Walter V Danilova L Robertson AG Johnson AR Comprehensive molecular characterization of pheochromocytoma and paraganglioma. Cancer Cell (2017) 31:181–93. 10.1016/j.ccell.2017.01.001 PMC5643159 28162975

[7] Burnichon N Vescovo L Amar L Libe R De RA Venisse A Integrative genomic analysis reveals somatic mutations in pheochromocytoma and paraganglioma. Hum Mol Genet (2011) 20:3974–85. 10.1093/hmg/ddr324 21784903

[8] Korevaar TI Grossman AB Pheochromocytomas and paragangliomas: assessment of malignant potential. Endocrine (2011) 40:354–65. 10.1007/s12020-011-9545-3 22038451

[9] Goffredo P Sosa JA Roman SA Malignant pheochromocytoma and paraganglioma: a population level analysis of long-term survival over two decades. J Surg Oncol (2013) 107:659–64. 10.1002/jso.23297 23233320

[10] Ziegler RG Weinstein SJ Fears TR Nutritional and genetic inefficiencies in one-carbon metabolism and cervical cancer risk. J Nutr (2002) 132:2345S–9S. 10.1093/jn/132.8.2345S 12163690

[11] Turkova H Prodanov T Maly M Martucci V Adams K Widimsky J Characteristics and outcomes of metastatic SDHB and sporadic pheochromocytoma/ paraganglioma: an National Institutes of Health study. Endocr Pract (2016) 22:302–14. 10.4158/EP15725.OR PMC7473461 26523625

[12] Hamidi O Metastatic pheochromocytoma and paraganglioma: recent advances in prognosis and management. Curr Opin Endocrinol Diabetes Obes (2019) 26:146–54. 10.1097/MED.0000000000000476 30893083

[13] Burnichon N Brière JJ Libé R Vescovo L Rivi`ere J Tissier F SDHA is a tumor suppressor gene causing paraganglioma. Hum Mol Genet (2010) 19(15):3011–20. 10.1093/hmg/ddq206 PMC2901140 20484225

[14] Astuti D Latif F Dallol A Dahia PL Douglas F George E Gene mutations in the succinate dehydrogenase subunit SDHB cause susceptibility to familial pheochromocytoma and to familial paraganglioma. Am J Hum Genet (2001) 69(1):49–54. 10.1086/321282 11404820 PMC1226047

[15] Niemann S Müller U. Mutations in SDHC cause autosomal dominant paraganglioma, type 3. Nat Genet (2000) 26(3):268–70. 10.1038/81551 11062460

[16] Baysal BE Ferrell RE Willett-Brozick JE Lawrence EC Myssiorek D Bosch A Mutations in SDHD, a mitochondrial complex II gene, in hereditary paraganglioma. Science (2000) 287(5454):848–51. 10.1126/science.287.5454.848 10657297

[17] Bayley JP Kunst HP Cascon A Sampietro ML Gaal J Korpershoek E SDHAF2 mutations in familial and sporadic paraganglioma and phaeochromocytoma. Lancet Oncol (2010) 11(4):366–72. 10.1016/S1470-2045(10)70007-3 20071235

[18] Suh YJ Choe J-Y Park HJ Malignancy in Pheochromocytoma or Paraganglioma: Integrative Analysis of 176 Cases in TCGA. Endocr Pathol (2017) 28:159–64. 10.1007/s12022-017-9479-2 28386672

[19] Antonio K Valdez MMN Mercado-Asis L Taïeb D Pacak K Pheochromocytoma/paraganglioma: recent updates in genetics, biochemistry, immunohistochemistry, metabolomics, imaging and therapeutic options. Gland Surg (2020) 9(1):105–23. 10.21037/gs.2019.10.25 PMC7082276 32206603

[20] Dahia PLM Pheochromocytoma and paraganglioma pathogenesis: learning from genetic heterogeneity. Nat Rev Cancer (2014) 14(2):108–19. 10.1038/nrc3648 24442145

[21] Turchini J Cheung VKY Tischler AS Krijger RRD Gill AJ Pathology and genetics of phaeochromocytoma and paraganglioma. Histopathology (2018) 72:97–105. 10.1111/his.13402 29239044

[22] Letouz´e E Martinelli C Loriot C Burnichon N Abermil N Ottolenghi C SDH mutations establish a hypermethylator phenotype in paraganglioma. Cancer Cell (2013) 23(6):739–52. 10.1016/j.ccr.2013.04.018 23707781

[23] Cascón A Comino-Méndez I Currás-Freixes M de Cubas AA Contreras L Richter S Whole-exome sequencing identifies MDH2 as a new familial paraganglioma gene. J Natl Cancer Inst (2015) 107(5):107. 10.1093/jnci/djv053 25766404

[24] Selak MA Armour SM MacKenzie ED Boulahbel H Watson DG Mansfield KD Succinate links TCA cycle dysfunction to oncogenesis by inhibiting HIFalpha prolyl hydroxylase. Cancer Cell (2005) 7(1):77–85. 10.1016/j.ccr.2004.11.022 15652751

[25] Latif F Tory K Gnarra J Yao M Duh FM Orcutt ML Identification of the von Hippel–Lindau disease tumor suppressor gene. Science (1993) 260(5112):1317–20. 10.1126/science.8493574 8493574

[26] Tella SH Taieb D Pacak K HIF-2alpha: Achilles’ heel of pseudohypoxic subtype paraganglioma and other related conditions. Eur J Cancer (2017) 86:1–4. 10.1016/j.ejca.2017.08.023 28946040 PMC6287501

[27] Zhuang Z Yang C Lorenzo F Merino M Fojo T Kebebew E Somatic HIF2A gain-of-function mutations in paraganglioma with polycythemia. N Engl J Med (2012) 367:922–30. 10.1056/NEJMoa1205119 PMC3432945 22931260

[28] Smestad JA Maher LJ Master regulator analysis of paragangliomas carrying SDHx, VHL, or MAML3 genetic alterations. BMC Cancer (2019) 19:619. 10.1186/s12885-019-5813-z 31234811 PMC6591808

[29] Welander J Soderkvist P and Gimm O Genetics and clinical characteristics of hereditary pheochromocytomas and paragangliomas. Endocr Relat Cancer (2011) 18:R253–76. 10.1530/ERC-11-0170 22041710

[30] Gimenez-Roqueplo A-P Dahia PL Robledo M An update on the genetics of paraganglioma, pheochromocytoma, and associated hereditary syndromes. Horm Metab Res (2012) 44:328–33. 10.1055/s-0031-1301302 22328163

[31] Neumann HP Bausch B McWhinney SR Bender BU Gimm O Franke G Germ-line mutations in nonsyndromic pheochromocytoma. N Engl J Med (2002) 346:1459–66. 10.1056/NEJMoa020152 12000816

[32] Burnichon N Buffet A Parfait B Letouzé E Laurendeau I Loriot C Somatic NF1 inactivation is a frequent event in sporadic pheochromocytoma. Hum Mol Genet (2012) 21:5397–405. 10.1093/hmg/dds374 22962301

[33] Qin Y Yao L King EE Buddavarapu K Lenci RE Chocron ES Germline mutations in TMEM127 confer susceptibility to pheochromocytoma. Nat Genet (2010) 42(3):229–33. 10.1038/ng.533 PMC2998199 20154675

[34] Burnichon N Cascón A Schiavi F Morales NP Comino-Méndez I Abermil N MAX mutations cause hereditary and sporadic pheochromocytoma and paraganglioma. Clin Cancer Res (2012) 18(10):2828–37. 10.1158/1078-0432 22452945

[35] Comino-Méndez I Gracia-Aznárez FJ Schiavi F Landa I Leandro-García LJ Letón R Exome sequencing identifies MAX mutations as a cause of hereditary pheochromocytoma. Nat Genet (2011) 43(7):663–7. 10.1038/ng.861 21685915

[36] Job S Draskovic I Burnichon N Buffet A Cros J Lépine C Telomerase Activation and ATRX Mutations Are Independent Risk Factors for Metastatic Pheochromocytoma and Paraganglioma. Clin Cancer Res (2019) 25(2):760–70. 10.1158/1078-0432.CCR-18-0139 30301828

[37] Letouze E Martinelli C Loriot C Burnichon N Abermil N Ottolenghi C SDH mutations establish a hypermethylator phenotype in paraganglioma. Cancer Cell (2013) 23:739–52. 10.1016/j.ccr.2013.04.018 23707781

[38] Yang C Zhuang Z Fliedner SM Shankavaram U Sun MG Bullova P Germ-line PHD1 and PHD2 mutations detected in patients with pheochromocytoma/paraganglioma-polycythemia. J Mol Med (Berl) (2015) 93:93–104. 10.1007/s00109-014-1205-7 25263965

[39] Calsina B Currás-Freixes M Buffet A Pons T Contreras L Letón R Role of MDH2 pathogenic variant in pheochromocytoma and paraganglioma patients. Genet Med (2018) 20(12):1652–62. 10.1038/s41436-018-0068-7 PMC7456538 30008476

[40] Buffet A Morin A Castro-Vega LJ Habarou F Lussey-Lepoutre C Letouzé E Germline Mutations in the Mitochondrial 2-Oxoglutarate/Malate Carrier SLC25A11 Gene Confer a Predisposition to Metastatic Paragangliomas. Cancer Res (2018) 78:1914–22. 10.1158/0008-5472.CAN-17-2463 29431636

[41] Remacha L Pirman D Mahoney CE Coloma J Calsina B Currás-Freixes M Recurrent Germline DLST Mutations in Individuals with Multiple Pheochromocytomas and Paragangliomas. Am J Hum Genet (2019) 104:1008–10. 10.1016/j.ajhg.2019.04.010 PMC6507037 31051110

[42] Sandoval JE Huang YH Muise A Goodell MA Reich NO Mutations in the DNMT3A DNA methyltransferase in acute myeloid leukemia patients cause both loss and gain of function and differential regulation by protein partners. J Biol Chem (2019) 294(13):4898–910. 10.1074/jbc.RA118.006795 PMC6442042 30705090

[43] Remacha L Currás-Freixes M Torres-Ruiz R Schiavi F Torres-Pérez R Calsina B Gain-of-function mutations in DNMT3A in patients with paraganglioma. Genet Med (2018) 20(12):1644–51. 10.1038/s41436-018-0003-y 29740169

[44] Remacha L Comino-Méndez I Richter S Contreras L María Currás-Freixes M Pita G Targeted Exome Sequencing of Krebs Cycle Genes Reveals Candidate Cancer-Predisposing Mutations in Pheochromocytomas and Paragangliomas. Clin Cancer Res (2017) 23(20):6315–24. 10.1158/1078-0432.CCR-16-2250 28720665

[45] Yeh I-T Lenci RE Qin Y Buddavarapu K Ligon AH Leteurtre E Germline mutation of the KIF1Bb gene on 1p36 in a family with neural and nonneural tumors. Hum Genet (2008) 124:279–85. 10.1007/s00439-008-0553-1 18726616

[46] Pillai S Gopalan V Lo CY Liew V Smith RA Lam AFK Silent genetic alterations identified by targeted next-generation sequencing in pheochromocytoma/paraganglioma: A clinicopathological correlations. Exp Mol Pathol (2017) 102(1):41–6. 10.1016/j.yexmp.2016.12.007 27986441

[47] Pang Y Gupta G Yang C Wang H Huynh T-T Abdullaev Z A novel splicing site IRP1 somatic mutation in a patient with pheochromocytoma and JAK2 V617F positive polycythemia vera: a case report. BMC Cancer (2018) 18(1):286. 10.1186/s12885-018-4127-x 29534684 PMC5850917

[48] Snezhkina AV Lukyanova EN Kalinin DV Pokrovsky AV Dmitriev AA Koroban NV Exome analysis of carotid body tumor. BMC Med Genomics (2018) 11(Suppl 1):17. 10.1186/s12920-018-0327-0 29504908 PMC5836820

[49] Welander J Łysiak M Brauckhoff M Brunaud L So¨derkvist P Gimm O Activating FGFR1 Mutations in Sporadic Pheochromocytomas. World J Surg (2018) 42:482–9. 10.1007/s00268-017-4320-0 PMC5762800 29159601

[50] Toledo RA Qin Y Cheng ZM Gao Q Iwata S Silva GM Recurrent mutations of chromatin-remodeling genes and kinase receptors in pheochromocytomas and paragangliomas. Clin Cancer Res (2016) 22(9):2301–10. 10.1158/1078-0432.CCR-15-1841 PMC4854762 26700204

[51] Wilzén A Rehammar A Muth A Nilsson O Tešan Tomić T Wängberg B Malignant pheochromocytomas/paragangliomas harbor mutations in transport and cell adhesion genes. Int J Cancer (2016) 138(9):2201–11. 10.1002/ijc.29957 26650627

[52] Tomić TT Olausson J Rehammar A Deland L Muth A Ejeskär K MYO5B mutations in pheochromocytoma/paraganglioma promote cancer progression. PloS Genet (2020) 16(6):e1008803. 10.1371/journal.pgen.1008803 32511227 PMC7329139

[53] Juhlin CC Stenman A Haglund F Clark VE Brown TC Baranoski J Whole-exome sequencing defines the mutational landscape of pheochromocytoma and identifies KMT2D as a recurrently mutated gene. Genes Chromosomes Cancer (2015) 54(9):542–54. 10.1002/gcc.22267 PMC4755142 26032282

[54] Hsu YR Torres-Mora J Kipp BR Sukov WR Jenkins SM Voss JS Clinicopathological, immunophenotypic and genetic studies of mediastinal paragangliomas. Eur J Cardiothorac Surg (2019) 56(5):867–75. 10.1093/ejcts/ezz115 31329844

[55] Papathomas TG Oudijk L Zwarthoff EC Post E Duijkers FA van Noesel MM Telomerase reverse transcriptase promoter mutations in tumors originating from the adrenal gland and extra-adrenal paraganglia. Endocr Relat Cancer (2014) 21(4):653–61. 10.1530/ERC-13-0429 24951106

[56] Dwight T Flynn A Amarasinghe K Benn DE Lupat R Li J TERT structural rearrangements in metastatic pheochromocytomas. Endocr Relat Cancer (2018) 25(1):1–9. 10.1530/ERC-17-0306 28974544

[57] Liu T Brown TC Juhlin CC Andreasson A Wang N Backdahl M The activating TERT promoter mutation C228T is recurrent in subsets of adrenal tumors. Endocr Related Cancer (2014) 21:427–34. 10.1530/ERC-14-0016 PMC4045219 24803525

[58] Irwin T Konnick EQ Tretiakova1 MS . Malignant Intrarenal/Renal Pelvis Paraganglioma with Co-Occurring SDHB and ATRX Mutations. Endocr Pathol (2019) 30:270–5. 10.1007/s12022-019-09594-1 31705439

[59] Zhang J Jiang J Luo Y Li X Lu Z Liu Y Molecular evaluation of a sporadic paraganglioma with concurrent IDH1 and ATRX mutations. Endocrine (2018) 61(2):216–23. 10.1007/s12020-018-1617-1 PMC7461619 29846902

[60] Fishbein L Khare S Wubbenhorst B Desloover D D’andrea K Merrill S Whole-exome sequencing identifies somatic ATRX mutations in pheochromocytomas and paragangliomas. Nat Commun (2015) 6:6140. 10.1038/ncomms7140 25608029 PMC4302757

[61] Yao L Barontini M Niederle B Jech M Pfragner R Dahia PLM Mutations of the Metabolic Genes IDH1, IDH2, and SDHAF2 Are Not Major Determinants of the Pseudohypoxic Phenotype of Sporadic Pheochromocytomas and Paragangliomas. J Clin Endocrinol Metab (2010) 95:1469–72. 10.1210/jc.2009-2245 PMC2841540 20130071

[62] Favier J Brière J-J Burnichon N Rivière J Vescovo L Benit P The Warburg Effect Is Genetically Determined in Inherited Pheochromocytomas. PloS One (2009) 4(9):e7094. 10.1371/journal.pone.0007094 19763184 PMC2738974

[63] Vicha A Taieb D Pacak K Current views on cell metabolism in SDHx-related pheochromocytoma and paraganglioma. Endocr Relat Cancer (2014) 21(3):R261–77. 10.1530/ERC-13-0398 PMC4016161 24500761

[64] Neumann HP de Herder W Energy and metabolic alterations in predisposition to pheochromocytomas and paragangliomas: the so-called Warburg (and more) effect, 15 years on. Endocr Relat Cancer (2015) 22(4):E5–7. 10.1530/ERC-15-0340 26273100

[65] Lu J Tan M Cai Q The Warburg effect in tumor progression: Mitochondrial oxidative metabolism as an anti-metastasis mechanism. Cancer Lett (2015) 356(2 Pt A):156–64. 10.1016/j.canlet.2014.04.001 PMC4195816 24732809

[66] Grasso D Zampieri LX Capelôa T Van de Velde JA Sonveaux P Mitochondria in cancer. Cell Stress (2020) 4(6):114–46. 10.15698/cst2020.06.221 PMC7278520 32548570

[67] van Berkel A Rao JU Kusters B Demir T Visser E Mensenkamp AR Correlation between in vivo 18F-FDG PET and immunohistochemical markers of glucose uptake and metabolism in pheochromocytoma and paraganglioma. J Nucl Med (2014) 55(8):1253–9. 10.2967/jnumed.114.137034 24925884

[68] van Berkel A Vriens D Visser EP Janssen MJR Gotthardt M Hermus ARMM Metabolic Subtyping of Pheochromocytoma and Paraganglioma by (18)F-FDG Pharmacokinetics Using Dynamic PET/CT Scanning. J Nucl Med (2019) 60(6):745–51. 10.2967/jnumed.118.216796 PMC6581230 30413658

[69] Katoh Y Katoh M Hedgehog target genes: mechanisms of carcinogenesis induced by aberrant hedgehog signaling activation. Curr Mol Med (2009) 9(7):873–86. 10.2174/156652409789105570 19860666

[70] Wilson NH Stoeckli ET Sonic Hedgehog regulates Wnt activity during neural circuit formation. Vitam Horm (2012) 88:173–209. 10.1016/B978-0-12-394622-5.00008-0 22391304

[71] Kobayashi H Kawauchi D Hashimoto Y Ogata T Murakami F The control of precerebellar neuron migration by RNA-binding protein Csde1. Neuroscience (2013) 253:292–303. 10.1016/j.neuroscience.2013.08.055 24012837

[72] Mihailovich M Militti C Gabaldon T Gebauer F Eukaryotic cold shock domain proteins: highly versatile regulators of gene expression. Bioessays (2010) 32:109–18. 10.1002/bies.200900122 20091748

[73] Dormoy-Raclet V Markovits J Malato Y Huet S Lagarde P Montaudon D Unr. A cytoplasmic RNA-binding protein with cold-shock domains, is involved in control of apoptosis in ES and HuH7 cells. Oncogene (2007) 26:2595–605. 10.1038/sj.onc.1210068 17086213

[74] Elatmani H Dormoy-Raclet V Dubus P Dautry F Chazaud C Jacquemin-Sablon H The RNA-binding protein Unr prevents mouse embryonic stem cells differentiation toward the primitive endoderm lineage. Stem Cells (2011) 29:1504–16. 10.1002/stem.712 21954113

[75] Schuchardt A D’Agati V Larsson-Blomberg L Costantini F Pachnis V The c ret receptor tyrosine kinase gene is required for the development of the kidney and enteric nervous system. Nature (1994) 367:380–3. 10.1038/367380a0 8114940

[76] Asai N Iwashita T Matsuyama M Takahashi M Mechanism of activation of the ret proto-oncogene by multiple endocrine neoplasia 2A mutations. Mol Cell Biol (1995) 3:1613–9. 10.1128/MCB.15.3.1613 PMC230385 7532281

[77] Santoro M Carlomagno F Romano A Bottaro DP Dathan NA Grieco M Activation of RET as a dominant transforming gene by germline mutations of MEN 2A. Science (1995) 267(5196):381–3. 10.1126/science.7824936 7824936

[78] Dahia PLM Clifton-Bligh R Gimenez-Roqueplo AP Robledo M Jimenez C HEREDITARY ENDOCRINE TUMOURS: CURRENT STATE-OF-THE-ART AND RESEARCH OPPORTUNITIES. Metastatic pheochromocytomas and paragangliomas: proceedings of the MEN2019 workshop. Endocr Relat Cancer (2020) 27(8):T41–52. 10.1530/ERC-19-0435 PMC7334096 32069214

[79] Thompson LDR Pheochromocytoma of the Adrenal Gland Scaled Score (PASS) to Separate Benign From Malignant Neoplasms A Clinicopathologic and Immunophenotypic Study of 100 Cases. Am J Surg Pathol (2002) 26(5):551–66. 10.1097/00000478-200205000-00002 11979086

[80] Kimura N Takayanagi R Takizawa N Itagaki E Katabami T Kakoi N Phaeochromocytoma Study Group in Japan. Pathological grading for predicting metastasis in phaeochromocytoma and paraganglioma. Endocr Relat Cancer (2014) 21(3):405–14. 10.1530/ERC-13-0494 24521857

[81] Wang Y Li M Deng H Pang Y Liu L Guan X The systems of metastatic potential prediction in pheochromocytoma and paraganglioma. Am J Cancer Res (2020) 10(3):769–80. PMC7136918 32266090

[82] Koh J-M Ahn SH Kim H Kim B-J Sung T-Y Kim YH Validation of pathological grading systems for predicting metastatic potential in pheochromocytoma and paraganglioma. PloS One (2017) 12(11):e0187398. 10.1371/journal.pone.0187398 29117221 PMC5678867

[83] Cho YY Kwak MK Lee SE Ahn SH Kim H Suh S A clinical prediction model to estimate the metastatic potential of pheochromocytoma/paraganglioma: ASES score. Surgery (2018) 164(3):511–7. 10.1016/j.surg.2018.05.001 29929757

[84] Pierre C Agopiantz M Brunaud L Battaglia-Hsu S-F Max A Pouget C COPPS, a composite score integrating pathological features, PS100 and SDHB losses, predicts the risk of metastasis and progression-free survival in pheochromocytomas/paragangliomas. Virch Archiv (2019) 474:721–34. 10.1007/s00428-019-02553-5 30868297

[85] Stenman A Zedenius J Juhlin CC. Over-diagnosis of potential malignant behavior in MEN 2A-associated pheochromocytomas using the PASS and GAPP algorithms. Langenbecks Arch Surg (2018) 403(6):785–90. 10.1007/s00423-018-1679-9 PMC6153590 29779047

[86] Gao B Meng F Bian W Chen J Zhao H Ma G Development and validation of pheochromocytoma of the adrenal gland scaled score for predicting malignant pheochromocytomas. Urology (2006) 68(2):282–6. 10.1016/j.urology.2006.02.019 16904437

[87] Kulkarni MM Khandeparkar SG Deshmukh SD Karekar RR Gaopande VL Joshi AR Risk Stratification in Paragangliomas with PASS (Pheochromocytoma of the Adrenal Gland Scaled Score) and Immunohistochemical Markers. J Clin Diagn Res (2016) 10(9):EC01–4. 10.7860/JCDR/2016/20565.8419 PMC5071941 27790441

[88] Wu D Tischler AS Lloyd RV DeLellis RA de Krijger R van Nederveen F Observer variation in the application of the Pheochromocytoma of the Adrenal Gland Scaled Score. Am J Surg Pathol (2009) 33(4):599–608. 10.1097/PAS.0b013e318190d12e 19145205

[89] van Nederveen FH Gaal J Favier J Korpershoek E Oldenburg RA de Bruyn EM An immunohistochemical procedure to detect patients with paraganglioma and phaeochromocytoma with germline SDHB, SDHC, or SDHD gene mutations: a retrospective and prospective analysis. Lancet Oncol (2009) 10(8):764–71. 10.1016/S1470-2045(09)70164-0 PMC4718191 19576851

[90] Castelblanco E Santacana M Valls J Cubas A Cascón A Robledo M Usefulness of Negative and Weak–Diffuse Pattern of SDHB Immunostaining in Assessment of SDH Mutations in Paragangliomas and Pheochromocytomas. Endocr Pathol (2013) 24:199–205. 10.1007/s12022-013-9269-4 24096807

[91] Zhou YY Coffey M Mansur D Wasman J Asa SL Couce M Images in Endocrine Pathology: Progressive Loss of Sustentacular Cells in a Case of Recurrent Jugulotympanic Paraganglioma over a Span of 5 years. Endocr Pathol (2020) 31(3):310–4. 10.1007/s12022-020-09632-3 32548761

[92] Kimura N Takekoshi K Naruse M Risk Stratification on Pheochromocytoma and Paraganglioma from Laboratory and Clinical Medicine. J Clin Med (2018) 7:242. 10.3390/jcm7090242 PMC6162838 30150569

[93] Sue M Martucci V Frey F Lenders JM Timmers HJ Peczkowska M Lack of utility of SDHB mutation testing in adrenergic metastatic phaeochromocytoma. Eur J Endocrinol (2015) 172(2):89–95. 10.1530/EJE-14-0756 25371406

[94] Konosu-Fukaya S Omata K Tezuka Y Ono Y Aoyama Y Satoh F Catecholamine-Synthesizing Enzymes in Pheochromocytoma and Extraadrenal Paraganglioma. Endocr Pathol (2018) 29(4):302–9. 10.1007/s12022-018-9544-5 30155766

[95] Deng L Chen T Xu H Li Y Deng M Mo D The Expression of Snail, Galectin-3, and IGF1R in the Differential Diagnosis of Benign and Malignant Pheochromocytoma and Paraganglioma. BioMed Res Int (2020) 27:4150735. 10.1155/2020/4150735 PMC7066411 32190664

[96] Leijon H Remes S Hagström J Louhimo J Mäenpää H Schalin-Jäntti C Variable somatostatin receptor subtype expression in 151 primary pheochromocytomas and paragangliomas. Hum Pathol (2019) 86:66–75. 10.1016/j.humpath.2018.11.020 30529752 PMC8192062

[97] Guo D Zhao X Wang A Xie Q Xu X Sun J Guo D PD-L1 expression and association with malignant behavior in pheochromocytomas/paragangliomas. Hum Pathol (2019) 86:155–62. 10.1016/j.humpath.2018.10.041 30594747

[98] Pinato DJ Black JR Trousil S Dina RE Trivedi P Mauri FA Programmed cell death ligands expression in phaeochromocytomas and paragangliomas: Relationship with the hypoxic response, immune evasion and malignant behavior. Oncoimmunology (2017) 6(11):e1358332. 10.1080/2162402X.2017.1358332 29147618 PMC5674959

